# An Innovative Neighbor Attention Mechanism Based on Coordinates for the Recognition of Facial Expressions

**DOI:** 10.3390/s24227404

**Published:** 2024-11-20

**Authors:** Cheng Peng, Bohao Li, Kun Zou, Bowen Zhang, Genan Dai, Ah Chung Tsoi

**Affiliations:** 1 School of Computing, Zhongshan Institute, University of Electronic Science and Technology of China, Zhongshan 528402, China; 2 School of Computer Science and Engineering, University of Electronic Science and Technology of China, Chengdu 611731, China; lbohao@163.com; 3 College of Big Data and Internet, Shenzhen Technology University, Shenzhen 518118, China; zhang_bo_wen@foxmail.com (B.Z.); daigenan@sztu.edu.cn (G.D.); 4 School of Computing and Information Technology, University of Wollongong, Wollongong, NSW 2522, Australia; moactsoi@gmail.com

**Keywords:** deep learning, facial expression recognition, neighbor attention model, coordinate attention

## Abstract

For solving the facial expression recognition (FER) problem, we introduce a novel feature extractor called the coordinate-based neighborhood attention mechanism (CNAM), which uses the coordinate attention (CA) method to capture the directional relationships in separate horizontal and vertical directions, the input features from a preprocessing unit, and then passes this to two residual blocks, one consisting of the neighborhood attention (NA) mechanism, which captures the local interaction of features within the neighborhood of a feature vector, while the other one contains a channel attention implemented by a multilayer perceptron (MLP). We apply the feature extractor, the CNAM module, to four FER benchmark datasets, namely, RAF-DB, AffectNet(7cls), AffectNet(8cls), and CK+, and through qualitative and quantitative analysis techniques, we conclude that the insertion of the CNAM module could decrease the intra-cluster distances and increase the inter-cluster distances among the high-dimensional feature vectors. The CNAM compares well with other state-of-the-art (SOTA) methods, being the best-performing method for the AffectNet(7cls) and CK+ datasets, while for the RAF-DB and AffectNet(8cls) datasets, its performance is among the top-performing SOTA methods.

## 1. Introduction

Humans communicate through speech, facial expressions, and pose. Facial expressions provide important information concerning a person’s state of mind, i.e., their emotions, and convey information on the underlying psychological state of the person [1].

In an online world, where images and videos are prevalent, it would be useful in a number of applications [2,3,4,5], e.g., human–machine communication [6], online shopping, internet commerce, social robots [7], to be able to recognize a person’s facial expressions and to gauge a person’s psychological state during the interaction.

It is recognized that facial expression recognition (FER) is a very challenging computer vision problem (see e.g., [5]. Broadly speaking, FER consists of seven or eight individual categories, e.g., happiness, anger, surprise, disgust, and fear; but there are also multi-level sub-categories, e.g., very happy, moderately happy, mildly happy, and slightly happier; and there are many compound categories, e.g., happily surprised, angrily surprised, etc. Anatomically, different facial expressions are expressed by minute adjustments of the facial muscles in different locations of the face simultaneously. Moreover, emotion can change abruptly and subtlely from one state to another. From a practical point of view, in an image, there may be more than one face and the face pose might not be aligned, or it may be tilted. The face might be self-occluded, in that if the face is rotated at an angle with respect to the horizontal axis, part of the facial features, e.g., the eye or cheek, may not be visible, or they may be occluded by other obstacles. The illumination under which the image was taken may also be far from ideal. In addition, the image quality might not be ideal; e.g., it may be blurry. A practical FER system would need to be able to overcome all these obstacles in order to be able to accurately recognize facial expressions.

In this paper, we are investigating a very simple version of this complex practical situation. We assume that the faces are captured with good illumination and are largely aligned, and most images contain only one face. Each image comes with a label, signifying the single category that a group of human experts have suggested as the most likely category of expression that the face in the image is expressing. Therefore, in the images that we considered, there is no landmark information—e.g., the locations of the pupils of the eyes, the locations of the eyebrows, the locations of the corners of the mouth—available; these examples are important in accurately recognizing facial expressions as they are where facial expressions are most visibly manifested. Moreover, as the labels are manually obtained by a group of humans, there will be issues with label noise. These are some of the assumptions behind the datasets which we studied (see [8,9,10]).

Traditionally, methods based on deep learning models (see e.g., [11,12,13]) for feature extraction were applied to study this classification problem.

This eventually gave way to vision transformer-based methods, e.g., the self attention mechanism and cross attention. This is because attention-based methods, are more adept in finding the long-range relationships between feature vectors.

Then, the importance of landmark information was popularized by [14,15], and this gave rise to some further work on incorporating landmark information into the model.

In this paper, we propose a novel deep learning-based model, called the coordinate-based neighbor attention mechanism (CNAM), based on two more advanced attention mechanisms, namely, coordinate attention (CA) [16] and neighborhood attention (NA) [17], as a postprocessing module for features extracted by two different feature extractors: IR50 [18], which extracts facial feature vectors with some mechanism to alleviate label noise in the dataset; and MobileFaceNet [19], which contains some information concerning landmarks as it is itself a postprocessing module for MTCNN [20], a method which could detect the locations of five facial landmarks: the pupils of both eyes, the tip of the nose, and both corners of the mouth. The preprocessing module IR60 [18] is selected as a way to minimize the unknown effects of the noise labels in the datasets, and MobileFaceNet [19] is selected as a convenient way to provide patch embedding of the landmark patches found in the MTCNN [20]. The CA module within CNAM could be conceived as modulating every pixel in every channel, with a function which is specific to the x-axis location and y-axis location of the pixel. These functions would be dependent on implicit landmark information, which is available from the features extracted by MobileFaceNet. This should be more effective than what some (see e.g., [21,22]) have recently used in modeling the directionality of the features obtained, say, from ResNet-18, through the deployment of an x-direction conv. kernel, e.g., 1×w, where w>1; and vertical-direction conv. kernel, e.g., w×1. The NA is a generalization of the self-attention mechanism in that it would compute the self attention of a query vector around a particular neighborhood and would thus be able to retrieve relevant values pertinent to that neighborhood. Thus, it is capable of using the feature vector at a particular location on the face image, and it can potentially retrieve information on its relationship in a neighborhood far away, relatively speaking, from a particular location.

We have implemented the CNAM method and have applied it to the following popular datasets: RAF-DB, AffectNet(7cls), AffectNet(8cls), and CK+. We found that in all cases, it achieves performances which would be among the very best, if not the best, of the state-of-the-art (SOTA) methods.

The main contributions of this paper are as follows:

- Our CNAM method is capable of making use of the landmark information embedded in features extracted from a preprocessor, e.g., MobileFaceNet [19], together with information again embedded in features extracted by a preprocessing module, which attempts to overcome the inherent noise label issues within a manually labeled training dataset, e.g., from IR50 [18,23], and obtains very respectable results on the following datasets: RAF-DN, AffectNet(7cls), AffectNet(8cls), and CK+.
- Our CNAM method, in comparison with other SOTA methods, achieves results which are among the very best—if not the best—results known at present.
- In using both qualitative and quantitative analysis techniques, e.g., confusion matrix analysis, grad-CAM, t-SNE, and three statistical indicators, namely, the Silhouette Coefficient (SC), the Davies–Bouldin Index (DBI), and the Calinski–Harabasz Index (CHI), we are able to obtain some insights into the behaviors of feature vectors after the insertion of CNAM; basically, the mean intra-cluster distance has decreased, while the inter-cluster distance has increased, and these have moved in the right direction after the insertion of the CNAM modules.

The following sections are organized as follows: Section 2 provides a brief description of some of the related works with respect to our CNAM method, while Section 3 provides a detailed description of the CNAM method. Section 4, after a presentation of the datasets used in this paper and the experimental setup, gives some qualitative and quantitative analysis results, a comparison of the CNAM method with other SOTA methods, and a description of the limitations of the CNAM method. Some conclusions are drawn and future research directions are provided in Section 5.

## 2. Related Work

In recent times, due to the fast advancement of demands in human–computer interaction, an increasing number of scholars have been directing their attention towards the recognition of human facial expressions. This is crucial for robots to comprehend humans. While conventional approaches, like employing manually designed features [24,25,26] for recognizing facial expressions, have been prevalent, they often struggle in difficult situations, such as in poor lighting conditions.

Recent studies in deep learning have raised questions about the relevance of handcrafted features. Two significant technologies for facial expression recognition are the visual attention mechanism and fine-grained visual recognition [27,28,29]. Visual attention mechanisms, as demonstrated in [30,31], have proven to be beneficial in improving the performance of various computer vision tasks such as image recognition and object detection. By mimicking the human visual system, these mechanisms allow computer vision systems to focus selectively on the most important regions or features in an image, thereby significantly improving their accuracy and efficiency. Fine-grained categories, as discussed in [32], often consist of numerous subcategories with subtle distinctions, which require extensive labeling efforts to capture discriminative features.

### 2.1. Visual Attention Mechanism

Visual attention is a crucial ability of the human visual system, enabling us to concentrate on the most significant aspects of a visual scene and disregard less crucial or extraneous details [33]. In the realm of computer vision, different attention mechanisms have been suggested by researchers, drawing inspiration from this ability to improve the efficacy of diverse tasks. This concept is applicable in the domain of expression recognition [34], as well as in tasks like object recognition, image captioning, and visual question answering.

One form of attention mechanism is the self-attention module, initially developed for machine translation [33]. This module determines the output at a specific position within a sequence by evaluating a weighted average across all positions. Xiao et al. expanded the application of self-attention to computer vision and associated it with non-local filtering techniques, introducing a broad non-local operation in deep neural networks [35]. Subsequent studies have expanded on this idea by creating an attention estimator that enhances the existing feature map [36,37].

### 2.2. Fine-Grained Visual Recognition

Distinguishing between objects within the same category that have subtle differences, like various species of birds or flowers, is a complex task in computer vision known as fine-grained visual classification. Conventional classification techniques [38,39,40] frequently struggle to capture the unique characteristics that set fine-grained categories apart.

To address this issue, modern approaches [41] integrate features at the part level, focussed attentive mechanisms, and semantic attributes to emphasize the most distinguishing areas and traits. They also utilize extensive sets of labeled data to capture nuanced signals that would be challenging to explicitly encode as crafted features. Additionally, certain techniques [42] utilize unsupervised pretraining methods to prepare models with a robust visual representation prior to supervised fine-tuning on a small amount of labeled data.

B-CNN [43] is a symmetrical convolutional neural network with two branches that calculates the outer product of its results to capture the second-order statistics of the convolutional feature maps. These descriptors of images can capture the relationships between different feature channels. Nonetheless, the high dimensionality of these descriptors can pose challenges for storage and further computation. Hence, several techniques, like CBP [44], LRBP [45], and DBT-Net [46], have been devised to lower the dimensionality of the features.

Attention mechanisms have demonstrated distinct benefits in detailed visual recognition tasks. For instance, Sermanet et al. integrated the attention mechanism into detailed recognition and presented a recurrent neural network model for acquiring visual attention [47]. Liu et al. expanded on this concept by employing a reinforcement learning approach to obtain visual attention [48]. Subsequent research, such as MA-CNN [29], MAMC [49], and WS-DAN [50], have further enhanced these approaches by formulating attention models in a systematic way, yielding highly encouraging outcomes on detailed recognition benchmarks.

Attention models have the capability to address issues related to misalignment in image matching and enhance the discriminative capacity of CNN features in the context of pedestrian and vehicle re-identification. For instance, Liu et al. and Lan et al. utilized attention models to pinpoint the prominent regions in images, thereby enhancing person re-identification [51,52]. Xu et al. and Zhao et al. developed a detector for human body parts that integrates the human body structure within the attention model [53,54]. Other approaches have applied attention mechanisms in video-based person re-identification to identify crucial segments in videos [55,56,57,58]. Khorramshahi et al. introduced an adaptive attention model that notably advanced the state of the art in tasks related to vehicle re-identification [59].

Items in a specific detailed category, like various types of birds, may exhibit noticeable variations in appearance because of factors like posture, lighting, and surrounding distractions. This substantial variation among items in the same category presents difficulties in developing visual representations and defining classification boundaries [60]. The challenge of detailed classification is akin to facial expression recognition, where discerning subtle distinctions in specific shape and texture variances is intricate and necessitates the identification of distinctive parts and features, a task that global approaches frequently find challenging to accomplish [61].

### 2.3. Facial Expression Recognition

In order to tackle the shared characteristics among various facial expression categories, different approaches have been developed. These strategies involve utilizing a modified version of the center loss [62] or a feature clustering network (FCN), which presents a simple expansion of the center loss [21].

With the rapid advancement of deep learning, many researchers have started employing convolutional neural networks for facial expression recognition (FER) tasks, achieving significant progress. Farzaneh et al. introduced the discriminant distribution-agnostic loss to control deep features in the embedding space, enabling the handling of extreme class imbalances as discussed in [63]. In their work [27], they utilized an integrated attention mechanism to determine the attention weight associated with the significance of features within the intermediate space feature map in the CNN, leading to improved facial expression outcomes. Xue et al. developed the TransFER model to acquire local facial emotional representations of various relational perceptions, as detailed in [64].

While many of these approaches can attain high accuracy under specific conditions, the technique introduced in this study is efficient and enhances the extracted data characteristics. This, in turn, strengthens the representation of data features, ensuring the method’s robust performance.

## 3. Method

### 3.1. Overview

The approach we have taken to solve this problem of image classification is as follows: given a set of labeled images, $T=\{I_{i},\ell_{i}\},i=1,2,\cdots ,N$, we wish to build a model M which will be able to predict the label of an image which is not in the T set. To make this a specific facial expression recognition problem, the images are known to consist of one or more faces, and the labels describe the state of the image: ‘happy’, ‘sad’, ‘angry’, etc. With the prior information that these are facial images, and that facial expressions are often simultaneously made up of minute muscle movements on facial landmarks, e.g., the corners of the mouth and the centers of the pupils, a successful model M would need to be able to capture the information which exhibits these invisible facial landmarks, which is invisible because these landmarks, while visible to humans, are not explicitly labeled as far as the computer is concerned. A model would need to be dependent on information in the neighborhood of a landmark would need to and infer from these landmark neighborhoods the most appropriate label with which to convey this information.

**Figure 1:**
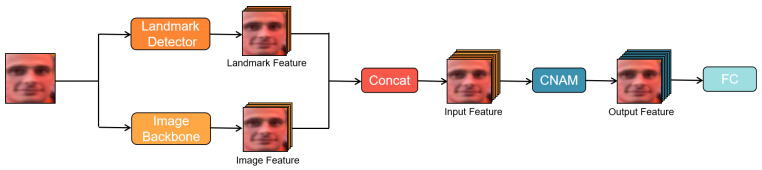
The overall architecture of our proposed model.

Our approach may be conveniently summarised into the following components: (1) preprocessing, in which two feature extractors are used, one a facial feature extractor and the other detects the location of five landmarks: the pupils on either eye, the tip of the nose, and the corners on either side of the mouth; (2) the coordinate attention (CA) module, in which the x and y coordinates of each channel are strengthened by the combined information along the x direction and the y direction; (3) the neighborhood attention (NA) module, in which the neighborhood information of each pixel is obtained; and (4) a postprocessing unit which consists of an MLP in which the information obtained so far is consolidated. These four components could be loosely said to comprise the feature extraction part, and the features obtained from this feature extractor are then classified using an MLP classifier. The model introduces a large number of trainable weights; these weights may be obtained through the minimization of a loss function (in our case, it is a cross entropy loss); and certain strategies, e.g., the DropPath strategy, in which randomly selected paths are not updated in the backward sweep of the backprop algorithm, use an appropriate learning algorithm, e.g., Adam, which has some means of estimating the second derivative of the loss function from the previous first-order derivatives. These need to be in place to minimize the risk of overfitting. However, our approach is an end-to-end optimization method, see Figure 1.

### 3.2. Preprocessing Unit

There are two parallel modules in the preprocessing Unit: an Ir50 feature extractor and a MobileFaceNet for landmark detection and feature extraction.

For an input image $H_{i}\times W_{i}\times 3$, the output of the Ir50 module is C×h×w, where h=w=7 and C=512, and the output of the MobileFaceNet is C×h×w. The e outputs of the Ir50 and MobileFaceNet are concatenated to form 2C×h×w, which is the output of the preprocessing unit.

#### 3.2.1. Coordinate Attention Module

The input 2C×h×w is summed along the x direction to form 2C×h×1, and the y direction to form 2C×1×w. The x direction component is then transposed to become 2C×1×h and concatenated with the y component to form 2C×1×(h+w).

This is then separated into two streams: 2C×1×h and 2C×1×w. One of these represents the horizontal (x) direction $f^{w}\in 2C\times w$, while the other represents the vertical (y) direction $f^{h}\in 2C\times h$. If $F_{h}\in R^{1\times 2C}$, and $F_{w}\in R^{1\times 2C}$, then

(1) $$\begin{array}{ccc} g^{h} & = & \sigma (F_{h}\left(f^{h}\right)), \end{array}$$

(2) $$\begin{array}{ccc} g^{w} & = & \sigma (F_{w}\left(f^{w}\right)), \end{array}$$

where $g^{h}\in R^{h\times 1}$ and $g^{w}\in R^{w\times 1}$. σ(·) is the sigmoid function.

The output of the coordinate attention block is as follows:

(3) $$y_{c}\left(i,j\right)=x_{c}\left(i,j\right)\times g_{C}^{h}\left(i\right)\times g_{C}^{w}\left(j\right),$$

where $x_{c}$ represents the *c*-th channel in the input, while $y_{c}$ represents the *c*-th channel of the output and c=1,2,⋯,2C.

In other words, the action of the CA would be to strengthen the (i,j)-th pixel in each channel (and there are 2C channels), which are strengthened by $g_{c}^{h}\left(i\right)$ and $g_{c}^{w}\left(j\right)$.

There are 2C×h×w outputs from the coordinate attention unit.

There is a residual connection from the input to the CA; therefore, the output would be 2C×h×w from the CA unit.

**Figure 2:**
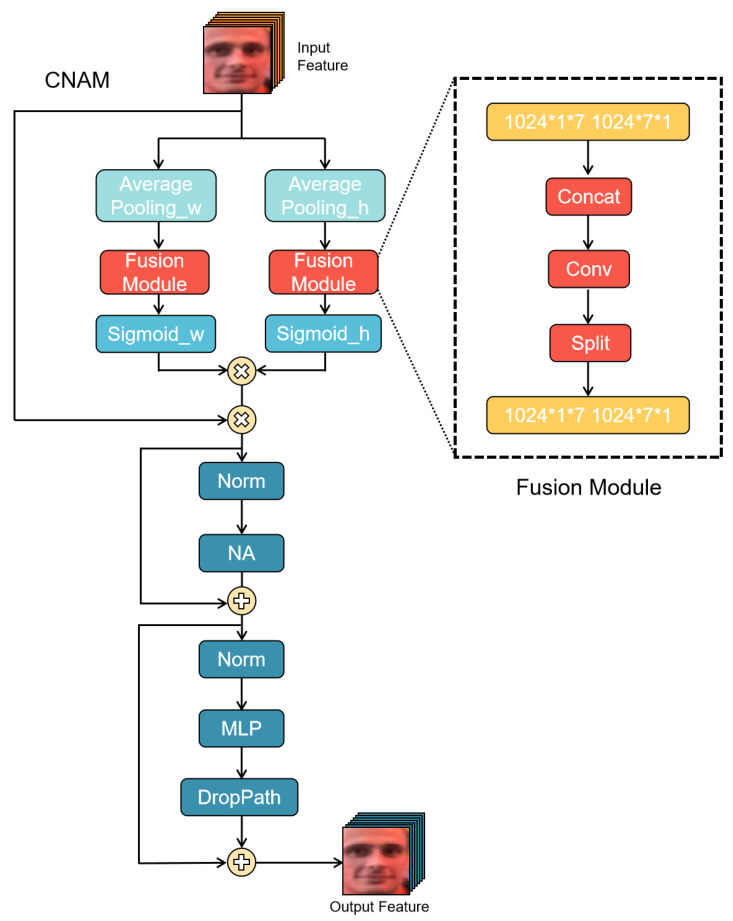
The pipeline of CNAM mainly contains the facial landmark detector, the image backbone, and vertical and horizontal feature processing.

This can be reduced to C×h×w through a simple transformation, see Figure 2.

#### 3.2.2. Neighborhood Attention Module

For the neighborhood attention (NA) module, we have the input $X\in R^{hw\times C}$. These are first linearly projected onto $Q=W_{Q}X$, $K=W_{K}X$, and $V=W_{V}X$. Here, $W_{Q}\in R^{hw\times hw}$, and $Q\in R^{hw\times C}$. Similarly, $W_{K}\in R^{\left(hw\times hw\right)}$, and $K\in R^{\left(hw\times C\right)}$. $W_{V}\in R^{\left(hw\times hw\right)}$, $V\in R^{\left(hw\times C\right)}$.

**Figure 3:**
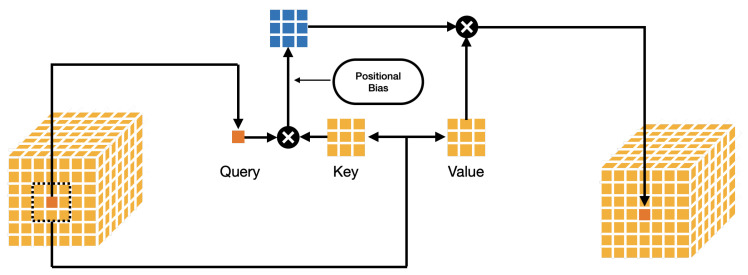
A figure illustrating the concept of neighborhood attention.

Neighborhood attention (NA) may be considered as a generalization of the self-attention (SA) mechanism in classic transformers [33]. It may be understood to be an SA for a single pixel (query) within a given neighborhood w×w, over which the key and value are formed. Thus, for SA, each pixel (query) attends to every other pixel, whereas for NA, it localizes attention to a neighborhood (w×w) only around itself. Figure 3 illustrates the concept of NA.

**Figure 4:**
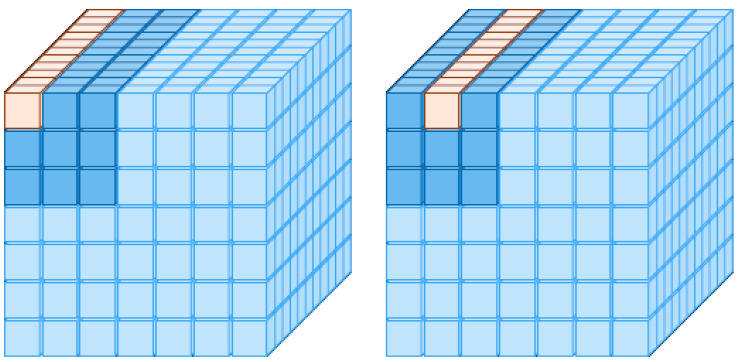
A figure illustrating the concept of NA. Here, the feature map is 7×7, and the neighborhood size is 3×3. The left hand figure depicts the pixel located at the 1,1 position as the query and its 3×3 neighborhood, while the figure on the right depicts the pixel at the 1,2 position as the query and its 3×3 neighborhood. The yellow-colored pixel indicates the location of the query pixel, while the deeper color indicates the 3×3 neighborhood.

For each pixel, as showed in Figure 4, we have a neighborhood w×w. Basically, one performs the self attention within this window, i.e., the QKV operation over this window of w×w. For example, if w=3, and a feature map is 7×7 for the first pixel, we wish to work out the 3×3 neighborhood, and so we have the following.

For the first 1×1 row of *Q*, as the query $Q_{11}$, the first 9 columns of the first 3×3 window of *K* and $K_{11}$, and the first 9 rows of *V* in the 3×3 window, $V_{11}$, we have

(4) $${NA}_{11}=softmax\left(\frac{Q_{11}K_{11}^{T}+B_{11}}{\sqrt{C}}\right)V_{11},$$

where $NA_{11}\in R^{\left(1\times C\right)}$ and $B_{11}\in R^{\left(1\times hw\right)}$ form a learnable bias for the $Q_{11}$ query vector.

Note that $Q_{11}$ is a corner element in the h×w space, and so does would require the padding by 0’s had we expanded the receptive field outside the h×w space; this is why we will make use of $K_{11}$ once more in the computation of ${NA}_{12}$.

$Q_{12}$ is a vector along the channel dimension, located at the 1,2-th element of *Q*.

(5) $${NA}_{12}=softmax\left(\frac{Q_{12}K_{11}^{T}+B_{12}}{\sqrt{C}}\right)V_{12}$$

Note that the $K_{11}$ is the same as the one for $Q_{11}$ because of edge effects. ${NA}_{12}\in R^{1\times C}$.

Proceeding in this manner through the *Q* matrix, we will have 49hw×C as the output. This is then reshaped to C×h×w.

This is then connected by residual connections with the input C×h×w to obtain C×h×w as the output of the NA module.

### 3.3. Postprocessing Unit

Each row of C×h×w is first re-shaped into 1×wC and then layer-normed and passed through an MLP with hidden layer width *C* and output 1×wC to obtain 1×wC. Then, it is assembled to form h×wC and then re-shaped to obtain C×h×w. A DropPath strategy is deployed to ensure that the risk of overfitting is minimized. This is connected by using residual connection with the input C×h×w to form C×h×w as the output of the postprocessing unit.

#### 3.3.1. MLP Classifier

The input here is C×h×w, and the output would be the predicted labels of the facial expression $\hat{\ell }$, where $\hat{\ell }$ is the predicted label of the facial expression as exhibit on the face.

This can easily be accomplished by an FC, with inputs C×hw and output 1×n. It could be two layers or one layer only.

#### 3.3.2. Cross Entropy Loss

The cross entropy loss is given by the following:

(6) $$\;mathcalL=-\sum_{i=1}^{N}t_{i}\log\left(p_{i}\right),$$

where $t_{i}$ is the target value and $p_{i}$ is the probability that $t_{i}$ will occur. *N* is the total number of labels. In practice, it is quite easy to obtain the $p_{i}$ if the output of the classifier goes through a softmax function.

For the unknown weights introduced in the CNAM, they can be obtained by minimizing the cross-entropy loss L.

One way of reducing the risk of overfitting is to use the DropPath strategy, which randomly selects the forward path and freezes the update of the selected path in the backward sweep of the backprop algorithm. We deploy the DropPath strategy in the postprocessing unit.

## 4. Experiments

In this section, after a description of the characteristics of the four datasets, namely, RAF-DB, AffectNet(7cls), AffectNet(8cls), and CK+ (see Section 4.1), the implementation details (see Section 4.2), and the details of an ablation study to determine the optimal size of the neighborhood in the NA module and the effect of having a CA module or not, which is part of the experimental setup, we will present the results of applying the CNAM method to these four datasets using a number of qualitative and quantitative analysis tools, namely, the confusion matrix analysis and visualization of the behaviors of the high-dimensional feature space using two different tools: grad-CAM [65] visualization of the influence of features extracted on the predicted category of an input facial image, and the t-SNE plots [66], which depict the clustering effects of the features at various locations of the CNAM method; and three statistical indicators: the Silhouette Coefficient (SC) [67], the Davies–Bouldin Index (DBI) [68], and the Calinski–Harabasz Index (CHI) [69], which shed light on the behaviors of extracted features at various locations in the CNAM method. This is followed by the comparison of the performance of CNAM with those of other state-of-the-art (SOTA) methods on each of the four datasets, followed by a discussion of some of the reasons why our CNAM perform well or not as well as in comparison with other SOTA methods. Finally, we discuss the limitations of the CNAM method and provide ideas as to how these may be alleviated in light of the comparisons with other SOTA methods.

### 4.1. Datasets

We assessed the facial expression recognition (FER) performance of CNAM on the RAF-DB, AffectNet(7cls), AffectNet(8cls), and CK+ datasets. The datasets’ configurations are summarized in Table 1.

- **RAF-DB** (Real world Affective Faces Database) [8] is a large-scale labeled facial expressions dataset. It comprises 315 individuals who are university students or faculty members, performing a range of expressions, such as smile, giggle, crying, anger, fear, shock, surprise, disgust, expressionless, surprise, happiness. The recorded images were subsequently labeled manually by crowdsourcing into seven classes: neutral, happy, sad, surprise, fear, disgust, and anger.
- **AffectNet** [9] is presently one of the most extensive publicly accessible dataset in FER, containing approximately 1 million facial images paired with labels which depict the underlying emotion of the faces in the images. Two datasets, AffectNet(7cls) and AffectNet(8cls), are extracted from this dataset, containing seven classes and eight classes of emotion, respectively. AffectNet(7cls) contains the following labels: neutral, happy, sad, surprise, fear, disguist, and anger; while AffectNet(8cls) contains an additional category: contempt in addition to those in the AffectNet(7cls).
- The **CK+ (Extended Cohn-Kanada) dataset** [10] is a small facial expression classification dataset. Images in this dataset are divided into seven classes: neutral, happy, sad, surprise, fear, disgust, anger. It is noted that this dataset is comparatively much smaller than the other three datasets.

### 4.2. Implementation Details

We implemented our experiments on GeForce RTX 4090 (NVIDIA, Santa Clara, CA, USA) using the Pytorch 2.0 framework. We used an IR50 network pretrained on the Ms-Celeb-1M dataset as the image backbone. MobileFaceNet, with fixed weights, was used as our face key point detector.

We used a batch size of 144, a learning rate of $3.5\times 10^{-4}$, and a weight decay of $1\times 10^{-4}$ and trained for 200 epochs on the training dataset. For the loss function, we used the cross-entropy loss. We used a random variable r∈U[0,1], where *r* is a random variable drawn from U[0,1], the uniform distribution between 0 and 1, to selectively freeze the fraction of the input path in the postprocessing unit in the backward sweep of the backprop algorithm. This is used as a regularization method to minimize the overfitting risk of the model.

### 4.3. Ablation Studies

In this section, we conducted two ablation studies: one using various sizes of the neighborhood for the NA module, and one concerning the key components of CNAM method, i.e., neighborhood attention and coordinate attention, which is divided into two components: horizontal (x direction) or vertical (y direction). The results are shown in Table 2.

#### 4.3.1. Size of the Neighborhood in the Neighborhood Attention Module

As the image is 7×7, it only makes sense to have a limited ablation study on this important hyperparameter. In this case, we choose two values: 1×1, 3×3, 5×5, or 7×7.

It is found that with a smaller neighborhood, 3×3 achieves the highest accuracy when compared with those of other neighborhood sizes. Therefore, we use a neighborhood of 3×3 in all our studies.

#### 4.3.2. The Effects of Having a Coordinate Attention Module

Table 2 shows the results for the CA module. It is observed that the accuracy degrades if the CA module is not present altogether (see the last row of Table 2). We used the NA with a neighborhood of 3×3, the result obtained from the ablation study above.

According to the results shown in Table 2, we will use both the NA module, with a neighborhood size of 3×3, and the CA module for all our studies.

It is worth noting that without the CA module, just using an NA module with the 3×3 neighborhood already achieves 90.25% accuracy, a figure which is comparable to the performances attained by most SOTA methods prior to 2022 (see Table 4 for details).

It is also interesting to note that CA appears to be more important than NA if one compares the first row (where NA is absent) with that of the last row (where CA is absent) in Table 4.

### 4.4. Qualitative and Quantitative Analysis of CNAM Method

In this section, we will provide some qualitative and quantitative analysis of the results of the application of our CNAM method on the four datasets.

#### 4.4.1. Confusion Matrices

**Figure 5:**
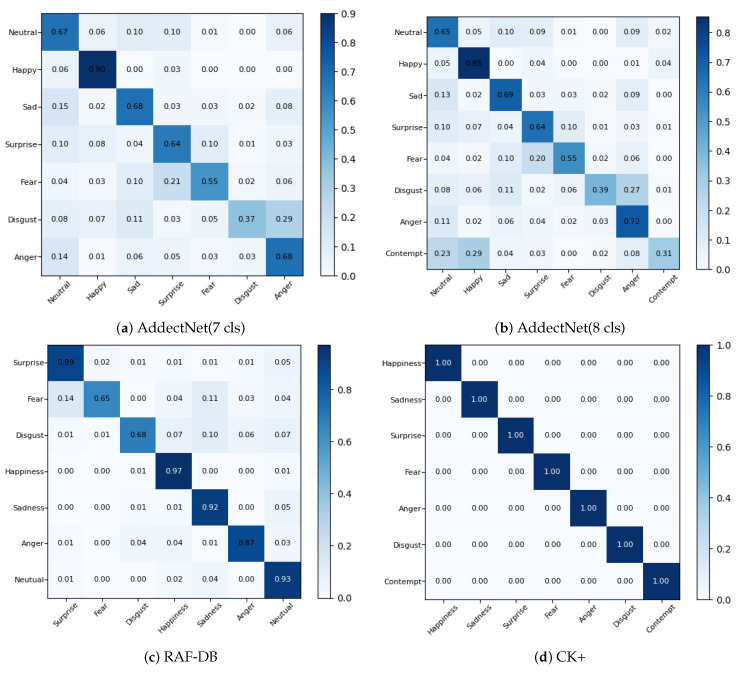
Confusion matrix analysis on AffectNet(7 cls), AffectNet(8 cls), RAF-DB, and CK+ datasets.

Confusion matrix analysis is a simple way to analyze the results of applying the CNAM method to the four datasets. Each element of the confusion matrix is usually computed as the ratio of the number of testing samples which fall into the predicted category versus the ground truth information. It is usually plotted with the ground truth categories as the x-axis and the predicted categories as the y-axis. Thus, a perfect 100% generalization accuracy, like the one representing the CK+ in Figure 5d, would be represented by a confusion matrix 1 along the diagonal and 0 for the off-diagonal elements. On the other hand, for AffectNet(7cls) or AffectNet(8cls), the confusion matrices would have diagonal elements, which might be small, while some of the off-diagonal elements might be quite large (see Figure 5a and Figure 5b, respectively). By viewing these confusion matrices (Figure 5), one can quickly grasp the accuracies of applying the CNAM method to the four datasets. For example, for the CK+ dataset, it scores a perfect 100% accurate on all categories; while, e.g., on the RAF-DB dataset, for an image with ground truth being in the “surprise” category, there is a 14% chance that it could be misclassified as “fear” (see Figure 5c for the elements in the confusion matrix).

It is possible to derive other statistical measures, e.g., precision, recall, F1 score, and AUC (area under the operation curve), from the confusion matrix. These can be understood as different ways of presenting the information contained in the confusion matrix.

#### 4.4.2. Heatmap Visualizations of Applying CNAM on the RAF-DB Dataset

**Figure 6:**
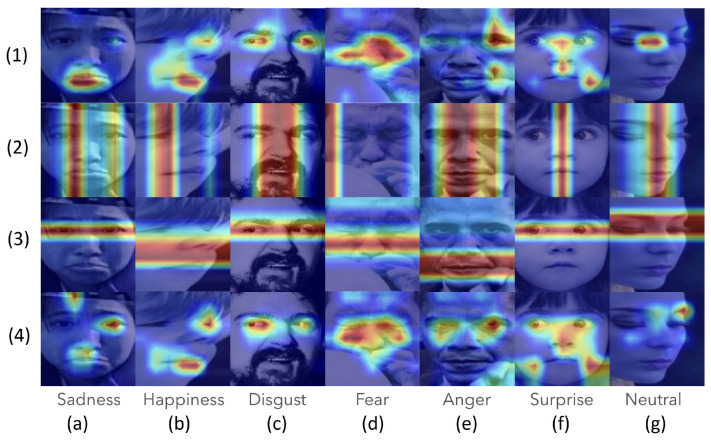
The correctly predicted samples from RAF-DB. Each subfigure may be referred to by their geographical location in this figure (**1a**–**4g**).

**Figure 7:**
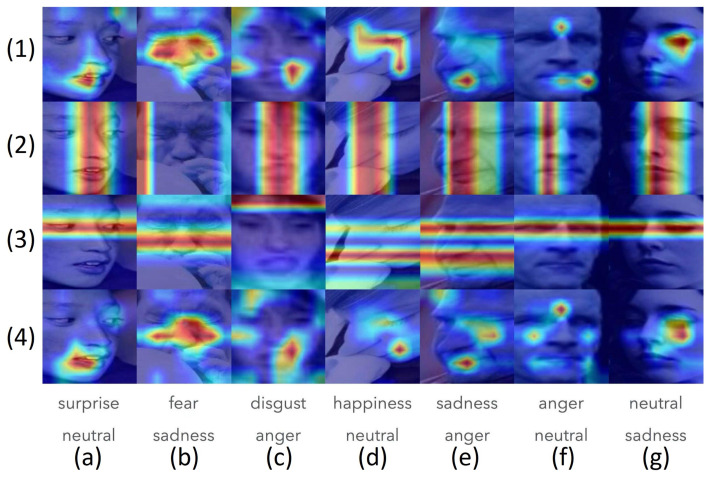
The wrongly predicted samples from RAF-DB, the up label is the true label and the bottom label is the predicted label (**1a**–**4g**).

The heatmap visualizations of the correctly predicted and incorrectly predicted labels in applying the CNAM method to RAF-DB are shown, respectively, in Figure 6 and Figure 7. The heatmap visualizations for the other datasets are omitted because they are quite similar to the ones shown in Figure 6 and Figure 7 for correctly predicted and incorrectly predicted images, respectively.

For Figure 6 and Figure 7, from the top to the bottom, the first row presents the visualization of the ResNet18 features; the second row, the vertical; the third row, the horizontal directions of the coordinate attention actions; and the bottom row, the neighborhood attention output. All visualizations were generated using grad-CAM [65], a gradient weighted class activation mapping approach, which is capable of highlighting the location of the highest (hottest) region (shown in red) on which the output label is based. Figure 6 shows samples that were predicted correctly, while Figure 7 shows samples that were predicted incorrectly. The last two lines of Figure 7 indicate, respectively, the correct category and the category in which the incorrect prediction was made.

The following observations are presented in order:

- It is observed in both figures (Figure 6 and Figure 7) that the ResNet-18 dataset is rather poor in that the hotspots identified might not be correct landmark locations (see Figure 6(1d), Figure 6(1e), Figure 6(1g); Figure 7(1b), Figure 7(1d)).
- In both Figure 6 and Figure 7, the second row shows the effect of the influence of the vertical direction, and the third row shows the influence of the horizontal direction of the coordinate attention, respectively. Due to the coarseness of the grad-CAM, it is rather more difficult to pinpoint what might have contributed to the coordinate attention in correctly or wrongly assigning the image to a certain category.
- The bottom rows of both Figure 6 and Figure 7 show the outcome of the CNAM after the neighborhood attention module. Again, due to the coarseness of the grad-CAM visualizations, it is rather difficult to draw hard and fast rules. It appears that in Figure 6, correct identification of the landmarks could have contributed to their prediction in the correct category; while bn contrast, in Figure 7, it appears that incorrect identification of the landmarks might have contributed to their being predicted to be in the wrong category.

#### 4.4.3. Feature Visualizations Using t-SNE Method

**Figure 8:**
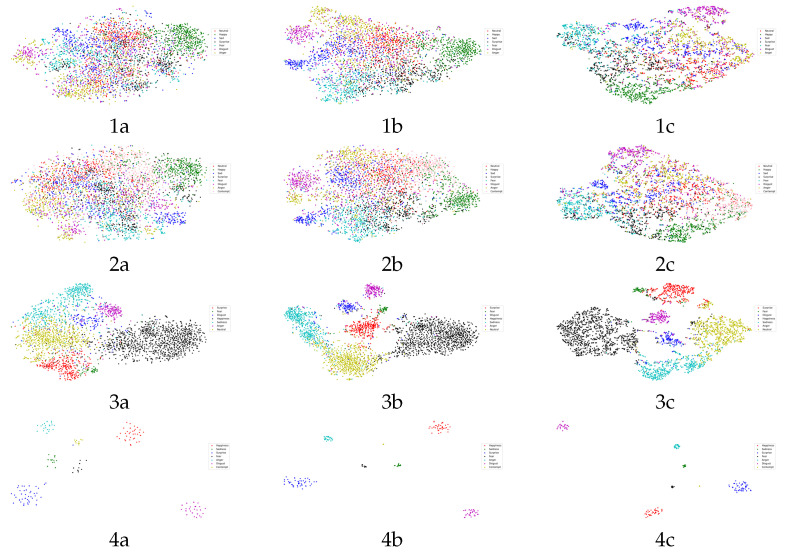
Visualization of t-SNE plots of AffectNet(7 cls) (**1a**–**c**), AffectNet(8 cls) (**2a**–**c**), RAF-DB (**3a**–**c**), and CK+ datasets (**4a**–**c**).

Figure 8 shows the visualization of the clustering results obtained by the t-SNE (t-stochastic neighborhood embedding) algorithm [66]. The t-SNE algorithm permits us to visualize the feature vectors in high-dimensional feature space on a two-dimensional display space [66]. The t-SNE plot may be used to visualize the effect of a processing module in increasing the separation of clusters. So, in our study, we used the t-SNE plot to visualize the features extracted by the preprocessing module (the IR50 model and the MobileFaceNet) after the CNAM module and the last fully connected layer. Note that the grad-CAM and the t-SNE plots are visualizing different aspects of those high-dimensional features: the grad-CAM considers the class upon which the features would have provided; i.e., it considers a predicted category at the output and seeks to find the patch in the feature space which might have been responsible for giving rise to this predicted category. For t-SNE, it considers the features as they are being projected onto a two-dimensional space for visualization. Thus, each technique attempts to visualize some characteristics of the high-dimensional set of features.

To understand the capabilities of t-SNE plots, it is perhaps academic to consider the last row in Figure 8 concerning the behaviors of the CK+ dataset. This is a small dataset, consisting of 327 samples over 7 classes in the training dataset. It is observed in Figure 8(4a), i.e., the t-SNE plot of the features after the preprocessing module, that seven clusters are clearly visible, though the purity of some of the clusters is not 1.0 (consisting of only one class in the cluster). Observe in Figure 8(4b) that after the CNAM module, these features are clearly in separate clusters, and clusters with impurity in Figure 8(4a) appear to have increased in size; thus, the classes which were previously well intertwined become better separated, thus making it easier for them to be separated by an MLP. Observe in Figure 8(4c) that all seven clusters are well separated, and all of them with a purity of 1; thus, it is little wonder that the generalization accuracy on the testing dataset achieves a perfect 100% score as these clusters can easily be separated with wide margins between the classes.

As to the other extreme, consider the case of AffectNet(8cls): it has 283,501 samples in the training dataset. Figure 8(2a) shows the t-SNE plot of the features after the preprocessing module. It is observed that the eight classes are well intertwined in the clusters and that the clusters are not separated at all. In Figure 8(2b), it is observed that some of the clusters are less intertwined, and some clusters appear to have much higher purities. This shows the ability of the CNAM in separating features that were previously much closer to one another. In Figure 8(2c), one observes that some of the clusters are further separated after the MLP functions, and the purities have further improved. The margins between classes are very close, thus providing little tolerance for the testing samples to be wrong.

It is observed that it is difficult to interpret the t-SNE plots for both the AffectNet(7cls) and RAF-DB, apart from the high-level observation that some of the clusters appear to have improved separation and purity after the CNAM module and the MLP module, respectively. However, it would be challenging to draw more information from these plots.

These examples show some of the challenges of using t-SNE, in addition to visualization of the data. It is challenging to try to interpret the t-SNE quantitatively, except in simple cases.

#### 4.4.4. Statistical Indicators

As we are dealing with feature vectors in a high-dimensional vector space, in the previous paragraph, we have shown that it is challenging to conclude anything concrete with respect to their behaviors using a projection of them onto a two-dimensional display space like t-SNE. One way of obtaining some quantitative measure is to use some statistical indicators, which will provide some information on their behavior in high-dimensional space. Out of a large number of possible statistical indicators, we choose three particular ones: the Silhouette Coefficient (SC) [67], the Davies–Bouldin Index (DBI) [68], and the Calinski–Harabasz Index (CHI) [69]. Each measures a particular aspect of the grouping of features in this high-dimensional space. All three indexes concern the quality of clusters formed, and each shows different aspects of these clusters in a high-dimensional space.

The SC [67] of a single sample is the ratio of the difference between the mean distance of a sample and all other points in the nearest cluster, to the mean distance between a sample and all other points in the same cluster divided by the max of these two measures. SC∈[−1,1], with a value of −1 for poorly formed clusters, while a value +1 indicates highly dense clusters. A value around 0 indicates overlapping clusters. A high value of SC implies that the cluster is well formed. The SC of a set of samples is the average of this quantity over the set. The Calinski–Harabasz index (CHI), also known as the variance ratio criterion, is defined as the ratio of the sum of between-cluster dispersion (the sum of distances squared) and of within-cluster dispersion for all clusters. A higher CHI indicates that the clusters are well defined. This score is higher when clusters are dense and well separated. Compared with SC, CHI could be unbounded, i.e., it could have large values, but it is much easier to compute. THe Davies–Bouldin Index (DBI) signifies the average similarity (a measure that compares the distance between clusters with the size of the clusters themselves) between clusters. The DBI is different from the SC or CHI in that its computation uses only point-wise distances. Zero is the lowest possible score. Values close to 0 indicate a better partition.

Armed with this information, the following observations can be made in Table 3.

- All three indexes give consistent results, indicating that the clusters formed by the features after the CNAM are better than those before the CNAM.
- For CK+, both the SC after CNAM (0.886) and the DBI after CNAM (0.1197) signify that the clusters formed by features prior to their entry to the MLP classifier are well formed, and well separated. This is corroborated by the t-SNE plot in Figure 8(4b). Therefore, an MLP classifier could easily provide 100% accuracy.
- For RAF-DB, SC after CNAM is higher than that before CNAM, and is is also ≈0.4, which indicates that the clusters are better formed than before CNAM. But the DBI is ≈1.5, which is considerably far from 0, indicating that the clusters are relatively well formed, though the purity of some of the clusters might be less than ideal. This provides some quantitative measures to the observation made in the t-SNE plot (see Figure 8(3b)). This is further confirmed in the confusion matrix (see Figure 5c); even after the MLP classifier, there are wrong predictiions pertaining to some of the categories, e.g.,there is a 0.14 probability that a “surprise” expression could be misclassified as “fear”.
- For AffectNet(7cls) and AffectNet(8cls), the SC scores are very close to 0, indicating that there are significant overlaps among the clusters. This could signify that it would be very difficult for the MLP classifier to correctly predict some of the samples. This is confirmed by the t-SNE plots (see Figure 8(1b) and Figure 8(2b), respectively). This is further collaborated by the confusion matrices of the respective datasets (see Figure 5a,b).
- Note that the CHI does not add much value except that it confirms the observations made on the SC and DBI.
- Please note that information conveyed by the confusion matrix, t-SNE, and the statistical indexes are statistical in nature; i.e., they cannot refer to a particular testing sample. For this, one would need to be dependent on the grad-CAM plot relating to an individual sample.

#### 4.4.5. Summary

In this section, we show that the confusion matrix, the t-SNE plot, and the three statistical indexes reveal different aspects of the statistical behaviors of CNAM on various datasets; while for individual samples, one would need to use grad-CAM to visualize the results and confusion matrix analysis to find the predicted category for that particular sample and whether it is correctly classified or not from the available ground truth information.

### 4.5. Comparison of the Performance of CNAM Method with Those Obtained by Other State-of-the-Art Methods

In this section, we will compare the performance of the CNAM with other state-of-the-art (SOTA) methods on the three datasets, namely, the RAF-DB, AffectNet(7cls), and AffectNet(8cls) datasets, and then on the CK+ dataset as the on the CK+ dataset, as noted in Section 4.4, CNAM achieved a perfect 100% score. Moreover, for RAF-DB, AffectNet(7cls), and AffectNet(8cls), we also compare the class-wise performance of CNAM with those obtained via other SOTA methods.

From the results shown in Table 4, Table 5 and Table 6. The performance of CNAM is among the very best of the SOTA methods. For RAF-DB and AffectNet(8cls), CNAM achieves results which are <1% of the current leader of the pack, while for AffectNet(7cls) and CK+, CNAM is the current leader of the pack. This success may be attributed mainly to the capability of CNAM in making use of the neighborhood information around each feature, as well as the global information as provided through the coordinate attention mechanism. In the following, we will make observations on comparing the performance of CNAM with other top-performing SOTA methods in an effort to determine what aspects of CNAM could be further improved by learning from the experience of these other top-performing SOTA methods.

#### 4.5.1. The Comparative Results on the RAF-DB, AffectNet(7cls), and AffectNet(8cls) Datasets

We compare the performance of our CNAM with those obtained by other SOTA FER methods on the RAF-DB, AffectNet(7cls), and AffectNet(8cls) datasets, and the results are shown in Table 4.

**Table 4:** Comparison results with state-of-the-art FER methods on RAF-DaB and AffectNet(7cls) and AffectNet(8cls). Numbers shown in red and blue, respectively, mark the highest and the second highest value in the same dataset. The figures under each dataset’s name denote the generalization accuracy expressed in %.

Methods	Years	RAF-DB	AffectNet(7cls)	AffectNet(8cls)
SCN [70]	CVPR2020	87.03	-	60.23
PSR [71]	CVPR2020	88.98	63.77	60.68
LDL-ALSG [72]	CVPR2020	85.53	59.35	-
RAN [73]	TIP2020	86.90	-	-
DACL [27]	WACV2020	87.78	65.20	-
KTN [74]	TIP2021	88.07	63.97	-
DMUE [75]	CVPR2021	89.42	63.11	-
FDRL [76]	CVPR2021	89.47	-	-
VTFF [77]	TAC2021	88.14	61.85	-
ARM [78]	2021	90.42	65.20	61.33
TransFER [64]	ICCV2021	90.91	66.23	-
DAN [21]	2023	89.70	65.69	62.09
EfficientFace [79]	AAAI2021	88.36	63.70	60.23
MA-Net [34]	TIP2021	88.42	64.53	60.29
Meta-Face2Exp [80]	CVPR2022	88.54	64.23	-
EAC [81]	ECCV2022	90.35	65.32	-
POSTER [14]	2022	92.05	67.31	63.34
POSTER-V2 [15]	2023	92.21	67.49	63.77
DDAMFN [22]	2023	91.35	67.03	64.25
ARBex [82]	2023	92.47	-	-
S2D [83]	2024	92.57	67.62	63.06
DCJT [84]	2024	92.24	-	-
BTN [85]	2024	92.64	67.60	64.29
FMAE [86]	2024	93.09	-	65.00
ours	-	92.37	67.63	64.14

The following observations may be made of Table 4:

- It is observed that the leader of the pack for RAF-DB and AffectNet(8cls) is FMAE (facial masked autoencoder) [86]. FMAE [86] is the first to use the following strategy: train a robust model on a masked augmented large dataset and then fine tune this robust model through an optimization process onto a relatively small dataset, like RAF-DB and AffectNet(8cls). It first created a large FER dataset through merging a number of existing FER datasets to obtain a Face9M dataset, which has approximately 9 million samples. For each sample, it uses the masked autoencoder (MAE) [87] to reconstruct the original image from a heavily masked input image, which can be trained in a self-supervised fashion. After the training has been completed, the lightweight decoder is discarded, and the trained encoder will be used in the fine tuning stage to adapt to a smaller dataset, like RAF-DB, through a fine tuning process of minimizing a simple classification loss, e.g., cross entropy loss. This does not involve any detection of landmark locations, as both RAF-DB and AffectNet(8cls) do not have any labeled landmark information. The success of this method may be attributed to two factors: very large training dataset of high resolution, and the ability of the MAE method in providing good and robust method with which to extract features.
  Fundamentally, FMAE works at the input image level, while CNAM, and many other methods, e.g., BTN, work as a postprocessing module; i.e., they process the information extracted by using some preprocessing steps to the input image. Therefore, in order to consider the idea of using an FMAE-like method, one needs to first overcome this fundamental issue. In the CNAM method, this can be easily achieved if we do not use the CA module but instead directly use the NA module to process the incoming image. So, it is possible to conduct the following experiments: first, we create a new large FER dataset by merging all the existing FER datasets. Let us denote this new large FER dataset as D. Then, we use an NA module as both the encoder and decoder in an MAE-like processing of D with heavy masks (up to 75% mask), trained in a self-supervised fashion. This will produce a robust pretrained NA model, which could be adapted to smaller datasets, e.g., RAF-DB, by fine tuning the pretrained model using a cross entropy loss, or other similar losses, which are used for classification purposes. Because the NA module processes information in a more sophisticated manner when compared with an autoencoder, it is highly likely that this method could produce new SOTA results.
- The second-best-performing method is BTN (batch transformer network) [85]. This is one of the few which recognize the importance of information within a batch of size *B*. It uses the same preprocessing step as CNAM, i.e., IR50 for feature extraction and MobileFaceNet for landmark features. Instead of using CA like CNAM, it processes the outputs from IR50 and MobileFaceNet in a multi-level fashion, i.e.,a pyramidal vision-like processing of the outputs, with the output from a lower level feeding as the input to a higher level; and then it combines these two multi-level outputs. Then, it processes this output using the batch transformer, which is essentially processing the information available in the batch, looking for the relationship between a particular query with features of the predicted classes in the batch. The success of this method currently exposes two weak points in our CNAM method: we do not process the CA in a multi-level manner, and we do not make use of the “free” information which is available in a batch. As indicated, we use a reasonably large batch size: 144, with 7 or 8 classes, and each class in a batch could consists of >15 images. A simple idea to extend CNAM would be to process the CA in a multi-level framework and then to divide the features according to their labeled information into *N* classes, where *N* is the number of categories in the dataset. We would then process the features in the same class as a neighborhood, and then one may use NA to obtain the relationships of one class of features with those of other classes. This is because in NA, the neighborhoods do not need to be contiguous, i.e., following one another; they could be considered just as a region in the feature space, and NA may be conducted on these discrete regions. As indicated above, FMAE uses a very crude way of processing the input image and does not make use of the inherent information concerning the landmark features; there is a real possibility that this more refined way of using CNAM could yield an even better accuracy than that provided by FNAE or BTN.
- It is interesting to observe the efficiency of CA when compared with other directional-based methods, e.g., DAN (distract your attention network) [21], DDAMEN (dual direction attention mixed feature network) [22]. CA is more effective in discovering the relationships between the horizontal and vertical features, which characteristize the human face, than the directional-based methods, because the directional-based methods use a directional convolutional kernel, while CA uses weighting in the horizontal or vertical directions (see, e.g., the grad-CAM visualizations in Section 4.4).
- It is instructive to consider the influence of the idea to incorporate landmark information in FER studies. Prior to the popularization of this idea by POSTER [14], the best methods, like DAN [21], do not use this information explicitly. But POSTER [14] and POSTERv2 [15] show that by using a pretrained OPenPose model to convey some rudimentary information concerning landmarks, the accuracy jumps by about 2%, which is a significant jump in this field. Since then, a number of papers, like S2D (static to dynamic) [83], BTN [85], and CNAM, further incorporate this landmark information in their methods and achieve SOTA results. This observation underlies one of the main reasons why we consider it to be important to incorporate this landmark information into FER. With an extention of FMAE, FMAE-IAL (FMAE–identity adversarial learning) [86], it is possible to make use of datasets which have ladnmark labels (such datasets are important in studying the challenging problem of FER in the wild, i.e., unaligned face images, as compared with those images in the four datasets used in this paper, which are largely aligned and center-cropped). Such labels are crucial to obtaining the best results on these landmark-labeled datasets.
- Methods like FMAE [86], BTN [85], and CNAM, S2D [83] do not consider the important issue: label noise in the datasets. This issue of label noise arises because the labels on the images in the four datasets are obtained manually. Despite our best efforts in attempting to eliminate the label noise issue, e.g., by crowdsourcing, e.g., RAF-DB [8], inevitably, there will be still some label noise in the datasets. There have been some attempts to address this important issue, such as Meta-Face2Exp [80] and EAC [81]. Probably because these were introduced prior to the idea that landmark information is important to FER, their results are not competitive when compared with those later methods like BTN [85], FMAE [86], or CNAM. However, it might be possible to incorporate some existing ideas of how to minimize label noise effect, like that of EAC [81], to improve the models are based on landmark location information, e.g., BTN [85] and CNAM.

#### 4.5.2. The Comparative Results on Class-Wise Classification in the RAF-DB, AffectNet(7cls), and AffectNet(8cls) Datasets

Table 5 shows the class-wise accuracy and mean accuracy of CNAM on the RAF-DB, AffectNet(7cls), and AffectNet(8cls) datasets.

Some observations based on Table 5 are as follows:

- As a general observation, the accuracies of some classes in the RAF-DB dataset are high, while for some other classes, they are not so high. For the AffectNet(7cls), they are all within a small band—around 60%—except for the category “Happy”. To some extent, this observation could also be made for the AffectNet(8cls), except that the figures are lower, e.g., for the “Happy” category; they are between 76% and 80%, while for other categories, they are less than those in the corresponding figures in the AffectNet(7cls) dataset. This could signify that images which are classified as “Happy” are easier to recognize, while images in the “Fear” and “Disgust” categories are relatively harder to recognize by the methods represented in Table 5. Indeed, this table is a simplified presentation of the results contained in the confusion matrix; i.e., these values are the diagonal values represented in the confusion matrices. From this, we may conclude that if CNAM finds it difficult make a classification, then the other methods also will also find it difficult, though the degree of difficulty could be different. Had the confusion matrices been available for all the methods, then it might have been possible to conclude the relative accuracies for the wrongly classified images among the categories in the dataset. Alternatively, some simple metrics, like FP (false positive rate), F1, or AUC, might reveal much more concerning the behaviors of the method at hand.

**Table 5:** Comparison of class-wise accuracy of CNAM and other state-of-the-art methods on RAF-DB, AffectNet(7 cls), and AffectNet(8 cls) datasets.

Dataset	Method	Accuracy of Emotions (%)								
		**Neutral**	**Happy**	**Sad**	**Surprise**	**Fear**	**Disgust**	**Anger**	**Contempt**	**Mean Acc (%)**
RAF-DB	MViT [88]	89.12	95.61	87.45	87.54	60.81	63.75	78.40	-	80.38
RAF-DB	VTFF [77]	87.50	94.09	87.24	85.41	64.86	68.12	85.80	-	81.20
RAF-DB	TransFER [64]	90.15	95.95	88.70	89.06	68.92	79.37	88.89	-	85.86
RAF-DB	POSTER [14]	92.06	97.22	92.89	90.58	68.92	71.88	88.27	-	85.97
RAF-DB	POSTER++ [15]	92.35	96.96	91.21	90.27	67.57	75.00	88.89	-	86.04
RAF-DB	APViT [89]	92.06	97.30	88.70	93.31	72.97	73.75	86.42	-	86.36
RAF-DB	BTN [85]	92.21	97.05	92.26	91.49	72.97	76.25	88.89	-	87.30
RAF-DB	CNAM	91.84	96.71	93.24	91.19	68.92	75.00	88.89	-	86.54
AffectNet(7cls)	APViT [89]	65.00	88.00	63.00	62.00	64.00	57.00	69.00	-	66.86
AffectNet(7cls)	POSTER [14]	67.20	89.00	67.00	64.00	64.80	56.00	62.60	-	67.23
AffectNet(7cls)	POSTER++ [15]	65.40	89.40	68.00	66.00	64.20	54.40	65.00	-	67.45
AffectNet(7cls)	BTN [85]	66.80	88.40	66.20	64.20	64.00	60.60	63.00	-	67.60
AffectNet(7cls)	CNAM	65.20	88.40	65.00	67.20	63.20	58.80	65.00	-	67.54
AffectNet(8cls)	POSTER [14]	59.40	80.20	66.60	63.60	63.60	59.80	58.80	54.71	63.34
AffectNet(8cls)	POSTER++ [15]	60.60	76.40	66.80	65.60	63.00	58.00	60.20	59.52	63.76
AffectNet(8cls)	BTN [85]	61.60	77.40	68.80	65.60	65.60	54.80	63.80	57.00	64.32
AffectNet(8cls)	CNAM	64.47	78.60	65.40	65.40	62.60	61.40	57.80	61.80	64.68

#### 4.5.3. The Results on the CK+ Dataset

We show the comparative performance of the CNAM method and other state-of-the-art methods on the CK+ dataset in Table 6.

CNAM achieved an accuracy of 100% on the CK+ dataset, outperforming all other methods. However, it is observed that other SOTA models also performed well on this dataset, achieving over 97% in the worst case shown in Table 6. As shown in the t-SNE plots in Section 4.4, for the features extracted by CNAM, they are well separated, and the inter-cluster distances are quite reasonable (a fact which is confirmed by the statistical indexes (see Section 4.4); therefore, it is little wonder that CNAM obtained 100% accuracy. While these analysis results are not available for other methods shown in Table 6, we would expect that they would show, to a large degree, similar behaviors to those observed in CNAM. The differences among these methods might just be due to their relative strength in extracting features. As to the results shown in Table 6, we may safely conclude that CNAM is comparatively a better feature extractor when compared with these other methods.

**Table 6:** Performance comparison on CK+ dataset, number shown in red marks the highest value.

Methods	Accuracy
SCAN-CCI [90]	97.31%
IF-GAN [91]	97.52%
Yanpeng Liu et al. [92]	98.30%
CNN-SIFT Aggregator [93]	99.10%
FDRL [76]	99.54%
Cornejo et al. [94]	99.78%
ViT + SE [95]	99.80%
**The proposed method**	**100.00%**

### 4.6. Limitations of CNAM

In summarizing our results, we discern the following limitations of CNAM, even though it achieves performances among the very top SOTA methods.

- CNAM may be considered a postprocessing module as it depends on the preprocessing models IR50 and MobileFaceNet. It is not a model which could directly process the image inputs. This is due to the fact that the CA module could only handle postprocessing duties from the landmark features provided by MobileFaceNet. It is possible to remedy this situation. If we take out the CA module and only have the NA module, in this case, the NA module would be able to process information directly from input images, e.g., rendering an image as a series of patches. But in this case, one would be disposing of the advantages of the CA module in processing the landmark features, which other SOTA methods, e.g., POSTERv2 [15], found useful. So, this would only make sense if one wishes to explore the possibility of FMAE [86] in the context of having access to a large composite FER dataset like Face9M [86].
- CNAM does not utilize the “free” information available in a batch as it uses a batch processing methodology. The “free” information within a batch could improve the CNAM performance further. So, how this batch information could best be exploited in a CNAM method would be an interesting direction for future research.
- CNAM does not explore the challenges posed by label noise, which are present, to some extent, in the FER datasets. This issue was highlighted when we applied CNAM to the CK+ dataset, which resulted in a generalization error of 100%, which could not be true in normal circumstances. This could be explained as purely fortuitous; the creators of this dataset happened to select a testing dataset which did not contain any label noise. Usually, however, one expects that there will be label noise in the data; therefore, it should be impossible to obtain 100% accuracy. It might therefore be interesting to explore some ways in which to alleviate label noise and incorporate it into CNAM.

## 5. Conclusions

In this paper, we introduce a novel deep learning model, CNAM, for studying FER. It uses a coordinate attention module followed by a neighborhood attention module. We provide a number of qualitative and quantitative analysis techniques: confusion matrix analysis, grad-CAM, t-SNE plot, and three statistical indexes, which measure some properties in the high-dimensional feature space. Moreover, we compare the performance of CNAM with those obtained by other SOTA methods and show that CNAM is among the top-performing FER methods, achieving the top spot on the AffectNet(7cls) and CK+ datasets, in particular.

There are a number of interesting directions for future research. One could explore the idea of using only the NA module in the context of the masked autoencoder (MAE) [87], and one could obtain access to much larger FER datasets, incorporate available information into a batch, and include strategies to alleviate label noise in the datasets. Developing strategies to alleviate label noise would be important if one wishes to explore the possibility of deploying methods like CNAM, or its improvements, to the FER of images in the wild as, in this context, the landmark labels are all manually annotated by human experts.

## Figures and Tables

**Table 1:** Detailed sizes of the datasets used in the experiments in this paper.

Datasets	Training Set Size	Testing Set Size	Classes
RAF-DB	12,271	3068	7
AffectNet(7 cls)	280,401	3500	7
AffectNet(8 cls)	283,501	3999	8
CK+	327	266	7

**Table 2:** Results of ablation experiments on two hyperparameters: the size of the neighborhood, i.e., if it is 1×1 or 3×3, 5×5, or 7×7 in the neighborhood attention module; and if a key component is present or not in the CNAM method on the RAF-DB dataset. Here, the coordinate attention is divided into two components: vertical (y direction) and or horizontal (x) direction. A ✓ means the component is present. CA indicates that it is the coordinate attention module, while NA denotes the neighborhood attention module.

Configuration					Accuracy %
**CA**	**NA (1×1)**	**NA (3×3)**	**NA (5 × 5)**	**NA (7×7)**	
${✓}^{1}$					90.71
✓	✓				91.72
✓		✓			92.37
✓			✓		91.59
✓				${✓}^{2}$	90.87
		✓			90.25

^1^: The NA module is bypassed by connecting its input directly to its output. CA is present. ^2^: An NA with a neighborhood the size of the image is reduced to the self attention mechanism.

**Table 3:** SC (Silhouette Coefficient), DBI (Davies–Bouldin Index), and CHI (Calinski–Harabasz Index) metrics depicting the behaviors of high-dimensional features on AffectNet(7cls), AffectNet(8cls), RAF-DB, and CK+ datasets. Higher SC and CHI values indicate better clustering performance, while lower DBI values indicate better clustering performance. Please see text for explanations as to what these indexes reveal of the behaviours of the features.

Dataset	Phase	SC ↑	DBI ↓	CHI ↑
RAF-DB	Before CNAM	0.2606	1.6819	1392.0486
	After CNAM	**0.3958**	**1.4916**	**1799.1164**
AffectNet(7cls)	Before CNAM	−0.0267	3.8635	295.1931
	After CNAM	**0.0581**	**3.1627**	**662.3302**
AffectNet(8cls)	Before CNAM	−0.0596	4.5313	318.6951
	After CNAM	**−0.0002**	**3.7724**	**614.1517**
CK+	Before CNAM	0.7543	0.2979	1516.6460
	After CNAM	**0.8860**	**0.1197**	**5420.1134**

↓ means the smaller the better and ↑ means the bigger the better.

## Data Availability

The original contributions presented in the study are included in the article, further inquiries can be directed to the corresponding author.
